# Platelets activated by the anti-β2GPI/β2GPI complex release microRNAs to inhibit migration and tube formation of human umbilical vein endothelial cells

**DOI:** 10.1186/s11658-018-0091-3

**Published:** 2018-05-15

**Authors:** Yanfen Zhang, Wenjing Zhang, Caijun Zha, Yanhong Liu

**Affiliations:** Department of Laboratory Diagnosis, The Second Affliated Hospital of Harbin Medical University, Harbin, China

**Keywords:** Platelet-derived microparticles, MicroRNA, Endothelial cells

## Abstract

**Background:**

Patients with anti-β2GPI antibodies display significantly higher platelet activation/aggregation and vascular endothelial cell damage. The mechanism underlying the correlation between platelet activation, vascular endothelial cell dysfunctions and anti-β2GPI antibodies remains unknown.

**Methods:**

In this study, we derived miR-96 and -26a from platelets activated by the anti-β2GPI/β2GPI complex and explored their role in modulating human umbilical vein endothelial cell (HUVEC) migration and tube formation.

**Results:**

Anti-β2GPI/β2GPI complex induces the release of platelet-derived microparticles (p-MPs). The amounts of miR-96 and -26a in these p-MPs were also higher than for the control group. Co-incubation of HUVECs with p-MPs resulted in the transfer of miR-96 and -26a into HUVECs, where they inhibited migration and tube formation. The targeting role of these miRNAs was further validated by directly downregulating targeted selectin-P (SELP) and platelet-derived growth factor receptor alpha (PDGFRA) via luciferase activity assay.

**Conclusion:**

Our study suggests that miR-96 and -26a in p-MPs can inhibit HUVEC behavior by targeting SELP and PDGFRA.

## Introduction

Antiphospholipid syndrome (APS) is characterized by thrombosis, pregnancy loss and the presence of antiphospholipid antibodies (APLAs). Anti-β2-glycoprotein I (anti-β2GPI) antibodies are the most common APLAs. They are directed against β2GPI [1], a phospholipid protein widely existing in the body. Anti-β2GPI antibodies play a central role in disease pathogenesis and may initiate the cascade of events that leads to platelet activation and thrombus development [2]. The mechanism underlying the activation of platelets by β2GPI and anti-β2GPI antibodies has been the focus of intensive research for decades.

Our previous study reported on anti-β2GPI-dependent platelet activation by LRP8 receptors and the mitogen-activated protein kinase 14 (MAPK14) pathway [3]. Anti-β2GPI antibodies are also known to activate human endothelial cells [4, 5]. The interaction of platelets and vascular endothelial cells and the gene regulation of these types of cells under the stimulation of the anti-β2GPI/β2GPI complex remain unclear.

Although platelets are anucleate and lack the ability to transcribe DNA, they are rich in cellular factors, including proteins and mRNAs involved in translation [6]. In recent years, several RNAs and their precursor forms, especially microRNAs and pre-miRNAs, have been identified in platelets [7, 8]. microRNAs are highly conserved small molecules consisting of 19 to 25 nucleotides of non-coding RNA that act as regulators of gene expression by targeting mRNA [9, 10]. Previous studies showed that platelets contain microRNAs that may come from megakaryocytes (miR-10a, − 126, − 150) [11, 12].

Cell-derived microRNAs have relatively recently been understood as gene regulators in human diseases [13, 14]. Subsequent studies suggested that some platelet activation factors, e.g., LPS or Ca^2+^, could induce the release of platelet microRNAs by platelet-derived tiny membrane vesicles, such as microparticles [15]. Previous studies showed that platelet miR-223 is delivered to lung cancer cells [16], endothelial cells [17, 18] or macrophages [19]. However, the role of anti-β2GPI antibodies in the release of platelet microRNAs in APS remains unknown.

Our results here show that the anti-β2GPI/β2GPI complex induces the release of platelet microparticles and increases the level of miR-96 and -26a in platelet-derived microparticles. The miR-96 and -26a derived from platelets inhibits the migration and tube formation of human umbilical vein endothelial cells (HUVECs). Finally, selectin-P (SELP) and platelet-derived growth factor receptor alpha (PDGFRA) were identified as the targets of these two miRNAs. Our study suggests that the anti-β2GPI/β2GPI complex regulates both platelets and vascular endothelial cells in the transfer of platelet-derived miRNAs.

## Materials and methods

### Platelet purification, activation and microparticle isolation

Platelets were isolated from venous blood and harvested by centrifugation at 1000×*g* for 10 mins and resuspended at 10^8^ platelets/ml in HEPES-tyrode buffer (pH 7.4) consisting of 130 mM NaCl, 3 mM KCl, 0.3 mM Na_2_HPO_4_, 12 mM NaHCO_3_, 20 mM HEPES, 5 mM monohydrate D-glucose and 0.5 mM MgCl_2_. All procedures were approved by the Institutional Medical Ethics Committee at Harbin Medical University.

Platelet activation and microparticle (MP) release were induced upon incubation with 0.1 U/ml of thrombin (Sigma-Aldrich) or anti-β2GPI/β2GPI complex for 60 min at 37 °C with gentle agitation. Anti-β2GPI/β2GPI complex (anti-β2GPI:β2GPI = 5/50 μg/ml or 10/100 μg/ml) was dissolved in Tris-buffered saline (TBS) as previously described [3].

The supernatant was centrifuged again to prepare a platelet-free release, which was used for MP isolation. MPs were harvested by centrifugation at 20,000×*g* for 90 min at 18 °C. A portion was resuspended in HEPES-Tyrode buffer for cell co-incubations. From the remainder, RNA was extracted through the addition of TRIzol (Invitrogen). The supernatant fraction was collected, snap frozen, and stored at − 80 °C until analysis.

### Transmission electron microscopy and flow cytometry

Microparticles obtained from activated platelets were resuspended and sent to the Electron Microscopy Center of Harbin Medical University for transmission electron microscopy analysis.

Microparticles were labelled with anti-human CD41-APC (BD Biosciences) for 20 min at room temperature in the dark per the manufacturer’s instructions. The samples were immediately analyzed using flow cytometry. To establish a microparticle gate, we used 500-nm size-calibrated beads (Sigma).

### miRNA analysis using quantitative RT-PCR

Total RNA was extracted from the platelet-derived microparticles. cDNA was synthesized using SuperScript II reverse transcriptase (Invitrogen Life Technologies) and a special reverse transcript primer (Table 1). Quantitative PCR was performed with a real-time PCR system using the primers shown in Table 1 with miRScript SYBR Green PCR Kit Mix (Qiagen). The values of miR-96, miR-26a and miR-26-3p were normalized to cel-miR-46*.

**Table 1:** Primers for detecting miR-96 and miR-26a based on the stemloop method

Primer name	Primer sequence
miR-26a-F	CCGGuucaaguaauccagga
miR-96-F	CCGGuuuggcacuagcacauu
cel-miR-46*-F	CCGGaagagagccgucuauu
miR-26a-R	CGTATCCAGTGCGTGTCGTG
miR-96- R	CGTATCCAGTGCGTGTCGTG
cel-miR-46*- R	CGTATCCAGTGCGTGTCGTG
miR-26a- Loop-RT-Primer	GTCGTATCCAGTGCGTGTCGTGGAGTCGGCAATTGCACTGGATACGAC *agccta*
miR-96- Loop-RT-Primer	GTCGTATCCAGTGCGTGTCGTGGAGTCGGCAATTGCACTGGATACGAC *agcaaa*
cel-miR-46*- Loop-RT-Primer	GTCGTATCCAGTGCGTGTCGTGGAGTCGGCAATTGCACTGGATACGAC *actgtc*

### Transfer of miRNA-FAM between platelets and HUVECs

The procedure for transfecting platelets was based on the protocol of Hong et al. [20]. Platelets were transfected with synthetic miR-96 or -26a mimics (Mimics are small artifical synthetic RNA molecules to mimic endogenous mature miR-96 or miR-26a) labelled with the carboxyfluorescein (FAM) fluorescent group. The MPs derived from the activated platelets were co-incubated with CD41 antibodies, and the transfer of miRNA was detected using fluorescence microscopy.

### HUVEC co-incubation with platelet-derived MPs

For MP transfer experiments, the platelet-derived MPs were centrifuged at 16,000×*g* for 30 min at 4 °C. The MP pellets were collected in sterile phosphate-buffered saline (PBS). HUVECs were incubated with platelet-derived MPs at a ratio of 1:100 (HUVECs: MPs) for up to 48 h at 37 °C, then the scratch wound and tube formation assays were performed.

### Scratch wound and tube formation assays

Migration of HUVECs was detected using a scratch wound assay. Briefly, the cells were then washed with the medium and co-cultured with platelet-derived MPs for 48 h, then the HUVECs were scraped with a sterile cell scraper to create a cell-free zone. Migration was photographed at the end of co-culture (0 h) and 24 and 48 h after scratching using an inverted microscope.

HUVECs co-cultured with platelet-derived MPs were cultured in 6-well plates coated with 200 μl of Matrigel Basement Membrane (BD Biosciences). Tube length was quantified with Image J software after 24 h by measuring the cumulative tube length in 5 random microscopic fields with an inverted microscope.

### miRNA target prediction and luciferase activity assay

The bioinformatics software microRNA.org was used to predict the miR-96 and -26a targets. The 3′ untranslated region (3’ UTR) of the target mRNAs were amplified via PCR from genomic DNA using cloning primers (Table 2). The amplicon was cloned into a pMIR-REPORT System (ABI). The nucleotides of the miR-96 or -26a seed sequence in the 3’ UTR of the targets were mutated using the QuikChange II Site-Directed Mutagenesis Kit (Agilent Technologies). The primers for the mutant construct are shown in Table 2. Each of these constructs was transfected into HEK293T cells together with 50 nM miRNA mimics or normal control (NC), and the pRL-TK vector (Promega) for the normalization of the transfection efficiency. The luciferase reporter assay was performed as described previously.

**Table 2:** The primers for SELP and PDGFRA wild-type UTR and mutant UTR

Primers	Sequences
Selp-CL-F:	tgtttcactagtttgggattgtggtac
Selp-CL-R	attctaagctttctgcagcctctggtg
Pdgfra-CL-F	gttggtactagttcattggcattctttgc
Pdgfra-CL-R	caaagctttgggaacaatgtaagtatc
Selp mutant UTR-F	gctttgactcacctgtagtctgaaataccagtgaaccaaagag
Pdgfra mutant UTR-F	gcagaagcaataataaagtggaagactacctactggtgtaatctc
	agacttggagaatacttggtaaacatttatgacaagctgtatcactgc
Selp mutant UTR-R	cagaactagcagaaacatttgctcctggcttc
Pdgfra mutant UTR-R	tcttccaaagcatcatctgccgatagcacag
	tatagaaatacatatatacatattgtat

### Western blotting

We transfected platelets with miRNA mimics or NC sequences, then extracted the platelet-derived MPs. Total protein from HUVECs co-cultured with MPs were extracted using radioimmunoprecipitation assay (RIPA) buffer. Protein expression was analyzed via western blotting. Glyceraldehyde 3-phosphate dehydrogenase (GAPDH; Santa) served as a loading control. Total protein extracts were separated using sodium dodecyl sulphate-polyacrylamide gel electrophoresis (SDS-PAGE) and transferred to polyvinylidene difluoride (PVDF) membranes. The level of SLEP, PDGFRA or GAPDH expression was evaluated using the appropriate antibodies (Santa), which were immunogen affinity purified. Bonds were quantified with Image-Pro Plus software.

### Statistical analysis

The data are expressed as means ± SD from at least three independent experiments. The differences between two groups in real-time PCR, scratch wound, tube formation and luciferase activity assay results were analyzed using two-tailed Student’s *t*-test. *p* < 0.05 is considered significant.

## Results

### Anti-β2GPI antibodies upregulate platelet microvesicles and platelet-derived miR-96 and -26a

**Fig. 1:**
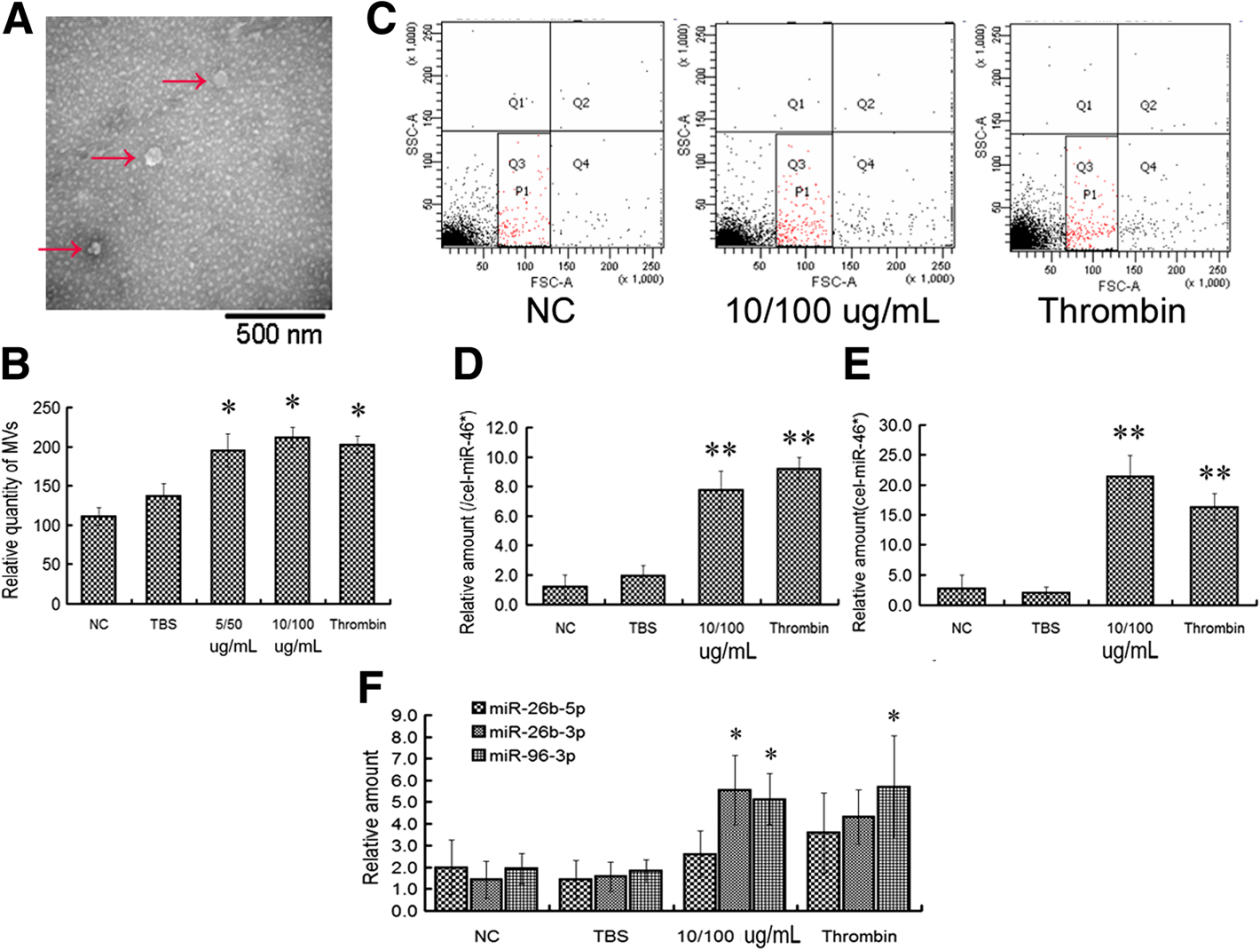
Anti-β2GPI/β2GPI complex induced the release of platelet-derived microparticles (MPs) and miRNAs. **a** Representative image of isolated MPs observed via transmission electron microscopy. Three MPs are indicated with arrows. **b** and **c** The amount of MPs with thrombin, the antibody complex or the control (TBS or NC) in the P1 area was detected using flow cytometry. MP gates were constructed via flow cytometry using size-calibrated beads. **d** and **e** The level of miR-96 (**d**) or miR-26a (**e**) in platelet-derived MPs was measured using quantitative RT-PCR. Cel-miR-46* was used as the internal control. **f** The relative levels of miR-26b-3p, −26b-5p and − 96-3p in the p-MPs were detected using quantitative RT-PCR. The results are presented as mean ± SD of four independent experiments (*p < 0.05, **p < 0.01 vs. TBS group)

MPs isolated from activated human platelets were detected using transmission electron microscopy. The MP diameters were between 30 and 50 nm (Fig. 1a). The MPs were counted and characterized via flow cytometry using the platelet glycoprotein CD41a surface antigen marker. The results show that anti-β2GPI/β2GPI complex increases the release of platelet-derived microparticles in a similar manner to thrombin (Fig. 1b, c). We chose the 10/100 μg/ml anti-β2GPI/β2GPI complex for subsequent experiments. The levels of platelet-derived miR-96 and -26a were higher after treatment with anti-β2GPI/β2GPI complex (Fig. 1d, e).

Only 2-fold changes in the levels of miR-96-3p, 26b-5p and 26b-3p (other members of the miR-26 family) after anti-β2GPI/β2GPI complex or thrombin treatment (Fig. 1f). Notably, the 10-fold increase in miR-96 and -26a was significantly higher than the increase in miR-96-3p and -26b-3p after the release of activated platelet-derived MPs.

These observations showed that the antibody complex induces the release of MPs from activated platelets and increases the amounts of miR-96 and -26a derived from platelets.

### Platelet-derived miR-96 and -26a stimulated by anti-β2GPI antibodies enter HUVECs

**Fig. 2:**
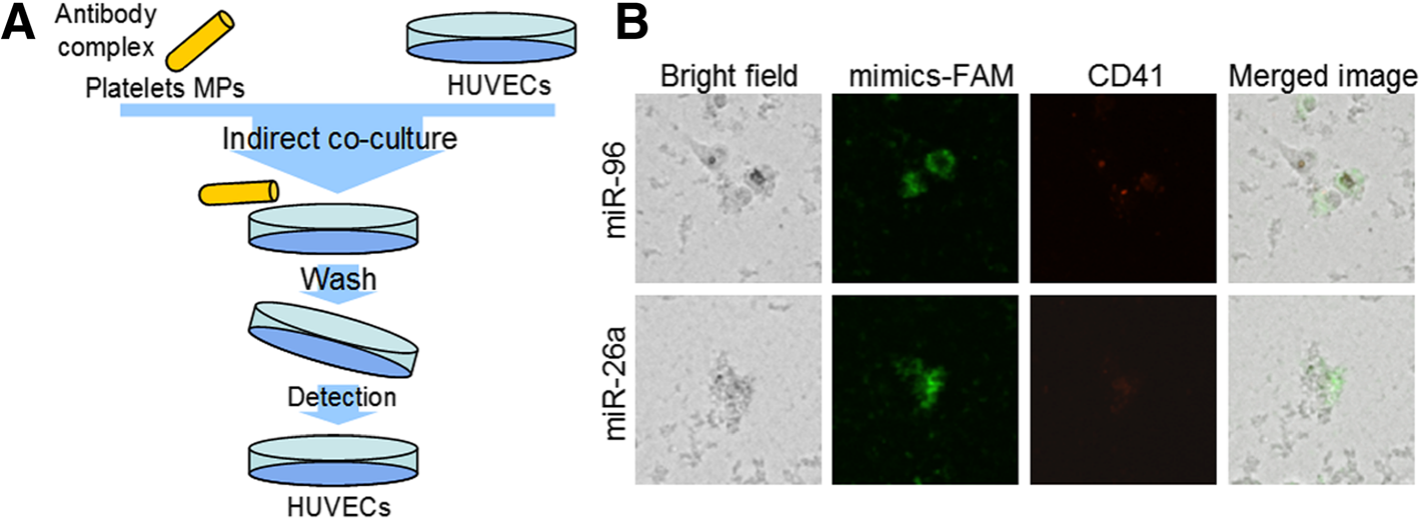
Platelet-derived miR-96 and miR-26a were transferred into HUVECs. **a** Illustration of co-incubation of platelet-derived microparticles (MPs) and HUVECs. **b** After the platelets were transfected with FAM-labelled miR-96 or miR-26a mimics, the MPs derived from platelets were incubated with CD41-APC (BD Biosciences) antibodies. 1.5 × 10^6^ HUVECs were seeded before the co-incubation of p-MPs and HUVECs (100:1). The signals of miR-96 or miR-26a are shown in green. The signals of CD41 are shown in red. The signals of miR-96 or miR-26a and CD41 are merged (Merged image)

Anti-β2GPI/β2GPI complex inducing the activation and aggregation of platelets and thrombosis has been reported previously [3]. Because cell-derived MPs act as signal molecule vectors between different cell types, we believe that platelet-derived microparticles might transfer to other cells in the circulatory system. Coagulation disorders not only result from the conditions of the platelets but also those of vascular endothelial cells, which are one of the most abundant cell types in the circulatory system. We performed platelet-derived MP–HUVEC co-incubation experiment to detect platelet-derived miR-96 and -26a MPs that could have been transferred into HUVECs. The results showed the miRNA-FAM signal (green) and CD41-APC signal (red) in HUVECs (Fig. 2).

### Platelet-derived miR-96 and -26a regulate vascular endothelial cells

**Fig. 3:**
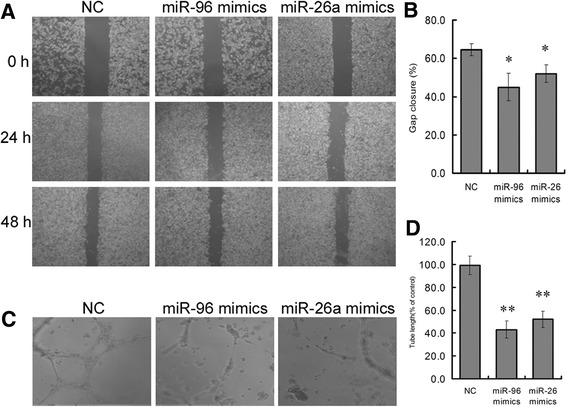
Overexpression of miR-96 or miR-26a inhibited migration and tube formation. **a** and **b** Migration of HUVECs co-incubated for 48 h with platelet-derived microparticles (MPs) after a scratch wound assay. **c** and **d** Tube formation was performed after co-incubation with p-MPs. Mimics are small RNA molecules to mimic endogenous mature miR-96 or miR-26a

To investigate the function of platelet-derived miR-96 and -26a in HUVECs, we transfected into platelets miR-96 or -26a using miRNA mimics and co-cultured them with microparticles from platelets and HUVECs for 48 h. We performed scratch wound and tube formation assays. The results indicated that activated platelet-derived miR-96 and -26a inhibited wound healing (Fig. 3a, b) and vesicular network formation (Fig. 3c, d) 48 h after scratching. These results suggest that the anti-β2GPI/β2GPI complex could regulate vascular endothelial cells directly and indirectly via platelet-derived microRNA.

### Platelet-derived miR-96 and -26a target p-selectin and vascular endothelial growth factor receptor a (VEGFRA) in vascular endothelial cells

**Fig. 4:**
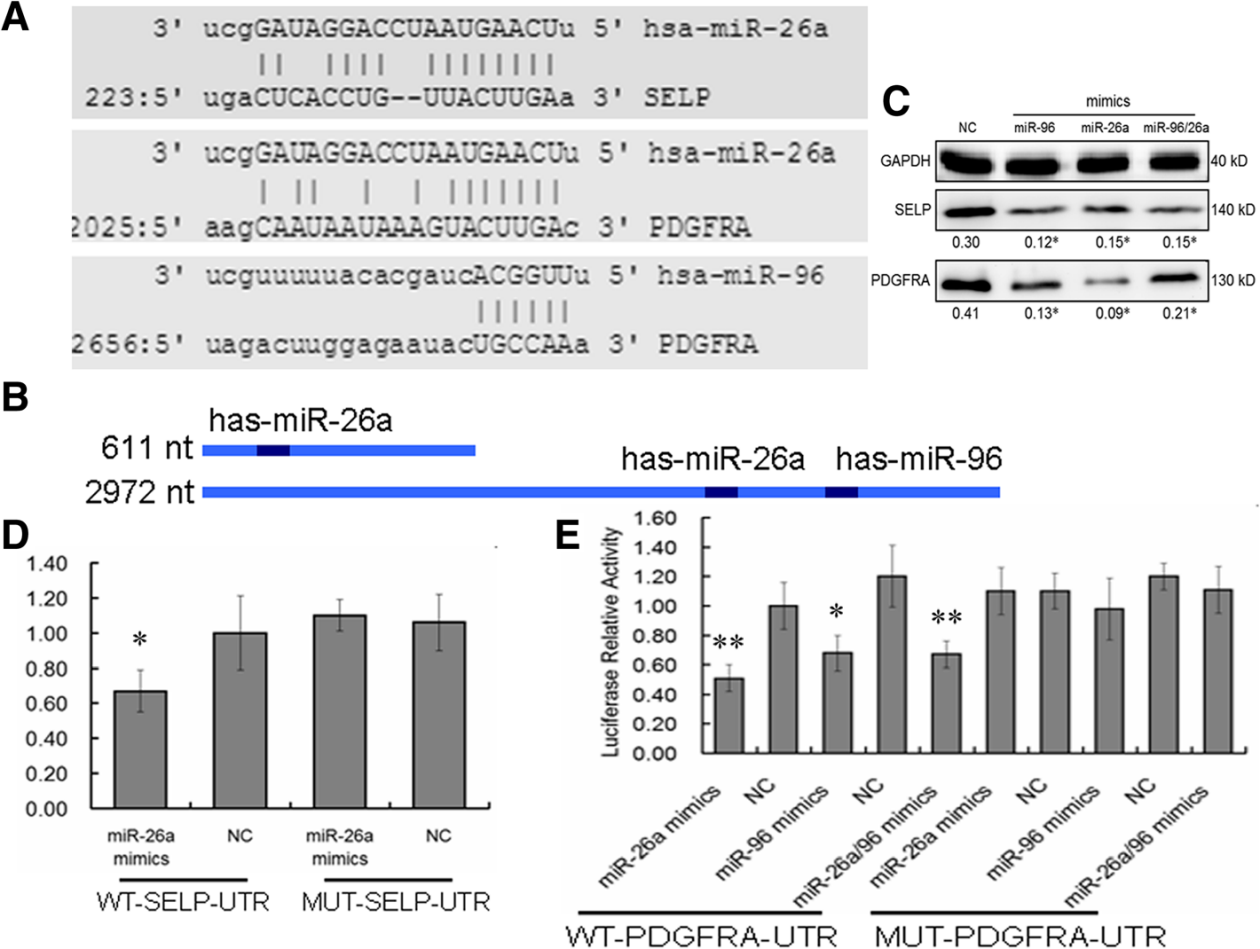
miR-96 and miR-26a downregulate SLEP and PDGFRA. **a** and **b** The sequence sites of miR-96 and miR-26a and the binding UTR of SLEP and PDGFRA. **c** miR-96 and miR-26a inhibit the expression of SLEP and PDGFRA at the translational level in HUVECs in vitro. The protein bonds are reduced in both groups. **d** and **e** The luciferase activity assay with the SLEP and PDGFRA UTRs. MiR-96 and miR-26a reduced the expression of SLEP and PDGFRA by targeting their UTRs. However, these miRNAs did not affect the activity of luciferase gene-containing mutant UTR. *p < 0.05, **p < 0.01). WT: wild-type (WT-UTR contains the binding site of miR-96 or miR-26a), MUT: mutant (MUT-UTR deletes the binding site of miR-96 or miR-26a)

The regulatory mechanism and targets for miR-96 and -26a have been reported in many tumors. However, their biologically functional targets in HUVECS remain unclear. To investigate these, we first predicted their targets using bioinformatics software (Fig. 4a). miR-96 and -26a were found to function by directly binding to the 3’ UTRs of their targets genes SELP or PDGFRA protein (Fig. 4b). Total protein from HUVECs co-incubated with platelet-derived miRNA mimics or NC were extracted. The expressions at the translational level were detected using western blotting (Fig. 4c). We performed a luciferase experiment to identify that miR-96 and -26a suppressed the expression of the targets by binding to 3’ UTR directly. When a wild-type UTR region of SELP or PDGFRA, containing miRNA response elements, was cloned into a luciferase report vector, the luciferase activity decreased by 38% (SELP) and 50 to 33% (PDGFRA). However, miR-96 and -26a did not reduce the luciferase activity when the construction contained SELP or PDGFRA mutant UTR (Fig. 4d, e).

## Discussion

Positive anti-β2GPI antibodies are frequently observed in patients with APS and systemic lupus erythematosus (SLE). Thrombosis and pregnancy loss are severe complications in patients with anti-β2GPI antibodies. The association between anti-β2GPI antibodies, platelet activation and vascular endothelial cell dysfunction was previously reported on, but the underlying mechanism remains unclear. It is known that abnormal thrombosis is a highly complex process resulting from both the state of the platelets and the states of other types of cells in the circulatory system.

Platelets have been reported to release the contents of α- or β-granules to other cells via exocytosis. Recently, many studies have reported that platelets could secrete MPs shedding from platelet membranes. Platelets are major sources of MPs in the peripheral bloodstream: two-thirds of blood MPs are likely derived from activated platelets [21]. Increasing evidence demonstrates that platelet-derived MPs (p-MPs) play a role in coagulation [22], angiogenesis [23] and cancer [24]. Increasing evidence suggests that platelets can affect the function and state of other types of cells by releasing p-MPs.

It was recently shown that large amounts of miRNAs exist in anucleate platelets [11, 12]. Here, we found that the levels of miR-96 and -26a in both platelets and p-MPs were higher after treatment with anti-β2GPI/β2GPI complex. The results showed that anti-β2GPI/β2GPI complex induced the release of p-MPs containing miRNAs, particularly miR-96 and -26a. These miRNAs could be delivered into recipient HUVECs via p-MPs. In the recipient cells, platelet miRNAs suppress the translation of their target genes, SLEP and PDGFRA. The ability of platelet-derived miR-96 and -26a to regulate the behavior of HUVECs might partially be due to the targeting of these genes.

This study provides more evidence supporting the concept that p-MPs serve as physiological carriers of functional miRNAs for exchanging genetic materials and signaling molecules between platelets and cells. To our knowledge, this is the first report of p-MP-derived miRNAs induced by the anti-β2GPI/β2GPI complex transferring between platelets and HUVECs.

The microRNA miR-96, which comes from an miRNA cluster, has been shown to cause dysexpression and dysfunction in many cells or cancers [25, 26], especially in platelets [27]. The study showed that miR-96 regulates VAMP8 expression and that this is responsible for the heterogeneity of platelet reactivity [27]. It was also found to regulate KRAS to suppress tumor cell invasion and migration [28]. MiR-26a, a member of the miR-26 family, has been found to exist in vascular endothelial cells and to target MAPK6, TRPC6 and BMP/SMAD1 signaling in angiogenesis in many diseases [29–31]. These miRNAs have been identified in vascular endothelial cells. However, their source has not been determined. Herein, we explored their transfer from activated platelets to HUVECs. Platelet-derived miR-96 and -26a might be another source of HUVEC miRNAs.

Happonen et al. reported that Gas6-Axl protein interaction could mediate endothelial cell uptake of p-MPs [32]. This study supported our conclusion that miR-96 and -26a could be transferred into HUVECs via p-MPs.

This study showed that the anti-β2GPI/β2GPI complex regulate HUVEC immigration and tube formation via platelet-derived miR-96 and miR-26a and that they target *SLEP* and *PDGFRA* mRNA in HUVECs. This novel interpretation suggests that anti-β2GPI/β2GPI complex regulates the function and state of HUVECs in patients with positive anti-β2GPI antibodies. The mechanism of dysregulation of miR-96 and miR-26a in activated platelets and the regulation of mature or alternative packaging remain to be further explored.

## Abbreviations

anti-β2GPI: Anti-β2-glycoprotein I
APLAs: Antiphospholipid antibodies
APS: Antiphospholipid syndrome
FAM: Carboxyfluorescein
GAPDH: Glyceraldehyde 3-phosphate dehydrogenase
HUVEC: Human umbilical vein endothelial cell
MAP K14: Mitogen-activated protein kinase 14
PDGFRA: Platelet-derived growth factor receptor alpha
p-MPs: Platelet-derived microparticles
RIPA: Radioimmunoprecipitation assay
SELP: Selectin-P
SLE: Systemic lupus erythematosus
